# Management Outcome of Burn Injury and Associated Factors among Hospitalized Children at Ayder Referral Hospital, Tigray, Ethiopia

**DOI:** 10.1155/2020/9136256

**Published:** 2020-02-18

**Authors:** Sielu Alemayehu, Bhafta Afera, Kalayou Kidanu, Tilahun Belete

**Affiliations:** Mekelle University, School of Nursing, Mekelle, Tigray, Ethiopia

## Abstract

**Background:**

Burn injuries are a global public health problem, accounting for an estimated 265,000 deaths. Globally, over half of the disability-adjusted life years lost from fire-related burns which occurred between the ages of 0 and 14 years. The rate of child deaths from burns is currently over 7 times higher in low- and middle-income countries than in high-income countries. In Ethiopia, burn was the second leading cause of death among children from the unintentional injuries. So far, no research had been conducted in terms of assessing the outcome of burn injury in children in Ethiopia and particularly in Tigray region. The aim of this study was to assess the outcome of burn injury and associated factors among hospitalized children of under 18 years at Ayder Referral Hospital in Mekelle, Ethiopia.

**Method:**

A retrospective document review was used to assess the outcome of burn injury and associated factors in Ayder Referral Hospital. A total of 382 hospitalized children's chart from 2011 to 2015 were reviewed using a structured check list. To select the patients' chart, a simple random sampling technique was used and a sampling frame was prepared based on a registration book. Data was entered, cleaned, and analyzed using SPSS version 20.

**Result:**

Almost 70% of the burns were caused by scald, and 45.3% of the burns were confined to the upper extremities. Eighty-two percent of the patients were discharged without complication. Lack of fluid resuscitation within 24 hours (AOR = 2.767; 95% CI (1.276-5.999)) and a burn patient with malnutrition (AOR = 0.252; 95% CI (0.069-0.923)) were statically significant with the outcome of burn injury.

**Conclusion:**

Majority of the pediatric burn patients were discharged without complication. The most causative agent of these accidents was scald; upper extremities also were the most affected area. The factors associated with the outcome of burn injury according to this study were lack of fluid resuscitation and malnourishment of burn patients.

## 1. Introduction

Burn injuries is a leading cause of unintentional injury, mortality, and morbidity. Globally, scalds account the highly treated burn that generally result in less severe injuries [1].

The high incidence of burn injuries in children is attributable to children's impulsiveness, lack of awareness, higher activity levels, natural curiosity, and total dependency on their caregiver [2]. In toddlers, the upper part of the body was the most frequently affected area [3], with children under the age of two years being at the highest risk for burns [4]. Caregivers were responsible for most of scald burns in infants [5]. Older children were more likely to receive burns from flame injuries [6].

Burn injuries are very common in developing countries, Burn victims typically come from poor families in rural regions, where fires are necessary for daily living and primary care is practically nonexistent [7]. Death by burn injury in low- and middle-income countries (LMICs) is estimated to be eleven times higher than in high-income countries as the World Health Organization (WHO) estimated that 43,000 people die of burns in Africa every year with a rate of 6.1 per 100,000 [8].

Most burns occur at home, causes being due to scald and flame [9]. Burn injuries are a global public health problem, accounting for an estimated 265000 deaths annually. The majority of these occur in low- and middle-income countries, and almost half occur in the Southeast Asia Region. The rate of child deaths from burns is currently over 7 times higher in low- and middle-income countries than in high-income countries. Burn trauma is an important public health concern, with increased risk for burns in children. Pediatric burns were more prevalent in developing countries and were associated with a high mortality rate [10].

Mortality due to burn is higher for children and the elderly. It accounts 65% scald injuries among under five children and 5-20 years old: 27% scald injuries. Nonaccidental burns are estimated to be as high as 20% of burn admissions. Inhalational injury increases mortality significantly [11].

Global estimates revealed that the highest number of pediatric burn admissions is found in the African continent. These injuries were rated as the second most common cause of accidental death in African children younger than 5 years of age [2]. In Africa, children under the age of 5 have almost 3 times the incidence of burn deaths than children worldwide. The burden of burn injury is highest among those children who live in poverty. In sub-Saharan Africa, it is estimated that between 18 000 and 30 000 children under the age of 18 die annually as a result of burn-related injuries [12].

Children have a relatively thinner dermis, so any given thermal insult will sustain a deeper burn than the adult [10]. Burn injury can impair skin integrity and sensation and lead to hypertrophic scarring. In addition to changes in appearance and function brought about scarring, deeper burns may result in damage to or complete loss of functionally or cosmetically important body parts. The impact of physical disfigurement due to burns is far reaching, as social stigma may lead to isolation and other psychological and physical impairments limit one's productivity. Children who sustain burn injuries may also develop posttraumatic stress disorder [13].

Annually, 3.2% of the South African population received burns with 50% occurring below the age of 20 years. Throughout Africa, the incidence of burns has increased due to poverty, illiteracy, urban migration, overcrowding, and the establishment of slum areas and shantytowns that have minimal safety measures and are generally unfit for human habitation. The majority of buildings were constructed from wooden frames and plastic materials that were susceptible to the rapid spread of fire [14]. In South Africa, burns are the third most common external cause of death in children younger than 18 years of age [15].

The risk of pediatric burn injuries in developing nations was primarily associated with the type of cooking substance and variety of cooking equipment used. Unstable pots and stoves were associated with a significant number of injuries. The incidence of childhood burn tended to be higher in rural children than in urban children [16]. In Ethiopia, burns were the third commonest unintentional injuries among children, next to firearms and falls [17]. Therefore, the aim of this study was to assess the management outcome of burn injury and associated factors among hospitalized children of under18 years at Ayder Referral Hospital in Mekelle, Ethiopia.

## 2. Materials and Methods

### 2.1. Study Area and Period

The study was conducted in Ayder Referral Hospital which is found in Mekelle city 783 km north of Addis Ababa with latitude and longitude 130 29′N39 ′E13.4830N39.4570E and at an elevation of 2084 meters above sea level. Mekelle city has nine governmental health centers, one referral hospital, one military hospital, and two general hospitals. Ayder Referral Hospital can be designated as the most advanced medical facility, by all accounts, in the northern part of the country. With the total capacity of about 500 inpatient beds in four major departments and other specialty units along with six other affiliated hospitals in the Tigray region, the burn unit of Ayder Referral Hospital is the only referral center in Tigray. Therefore, the patients presenting to this unit are generally from almost all regions of Tigray, at the cross roads between Afar and Amhara. The study was conducted from February to June 2016.

#### 2.1.1. Study Design

A retrospective facility-based document review analytical study design was used.

#### 2.1.2. Source Population

The source population for this study was all children under 18 years of age who were admitted because of burn injury at Ayder Referral Hospital from 2011 to 2015.

#### 2.1.3. Study Population

The study population for this study was all selected patient's chart of children under 18 years of age who were admitted because of burn injury at Ayder Referral Hospital from 2011 to 2015.

#### 2.1.4. Inclusion Criteria

The inclusion criteria include all selected patient's chart of children of under 18 years of age with burn injury, which was with complete medical record.

#### 2.1.5. Exclusion Criteria

The exclusion criteria include children who were admitted in a burn unit but left against medical advice (LAMA).

#### 2.1.6. Sample Size Determination

Considering the time and economic feasibility, sample size calculation was considered. The sample size was first calculated using a single proportion formula assuming the management outcome of burn injury from Yekatit 12 Hospital in which 49% of the children with burn injury had developed contracture (*P* = 0.49) [14]. This outcome was used because it gave the highest possible sample size compared to the other outcomes. 95% confidence interval and 5% significance level were also considered in the following formula:

(1) $$\begin{array}{c} n=\frac{{\left(Z\alpha /2\right)}^{2}P\left(1-P\right)}{d^{2}},\\ {\left(1.96\right)}^{2}\left(0.49\right)\left(1-0.49\right)=384, \end{array}$$

where *n* is the minimum sample size required, *z* is the standard score corresponding to 95% confidence interval, *P* is the proportion of the outcome of pediatric burn (49% in Yekatit 12 Hospital), and *d* is the margin of error (precision) (5%).

Then by using *n* = 384 from the above and *N* = total admitted pediatric patients from registration book of Ayder Referral Hospital, the correction formula was used to get the final sample size:

(2) $$\begin{array}{c} nf=\frac{n}{1}+\frac{n}{N},\\ nf=\frac{384}{1}+\frac{384}{7605},\\ nf=365. \end{array}$$

With 5% contingency for illegible handwriting, so 382 patients' medical record cards were included for the final analysis.

*(1) Sampling Technique*. There were a total of 1631 children admitted in the hospital in 2011 to 2015.

The medical chart sampling frame was prepared based on a medical record in the hospital's patent registration book; considering the economic constraints and the time given by the institution to finalize the research, patients' chart was selected from the sampling frame using systematic random sampling considering every 4^th^ medical chart.

*(2) Data Collection Procedures*. Based on their patient's medical record number, the children's chart was traced. The data collection process was carried out. Additional information of pediatric patients was collected from their register. Three (nurses) data collectors were trained for half day, and one supervisor was recruited to undertake the data collection process; the principal investigator was followed the overall procedures.

*(3) Data Collection Instrument*. A structured checklist was prepared in English from the chart and from the questionnaire of the study done in Addis Ababa [14], and the checklist contains close-ended questions. Tools and materials mainly measure sociodemographic characteristics, outcome of burn injury, and risk factors associated with outcome of burn resulted from different reasons.

*(4) Data Management and Analysis*. Data was entered and analyzed using SPSS 20. Descriptive statistics was computed for independent variables and outcome variable. Categorical variables were presented in the form of frequencies and percentages. Texts, tables, and graphs were used to present the results. Binary logistic regressions were used to analyze the relationship between the dependent and independent variables. Bivariate analysis was computed; then all explanatory variables with *P* value of <0.2 were entered into multivariable logistic regression. Association was done using odds ratio; *P* value < 0.05 was considered statistically significant.

*(5) Data Quality Control*. To assure the quality of the data, emphasis was given in designing the data collection instruments based on the patient chart/registration. Half day training was given to data collectors and the supervisor on how to collect data from the chart. During the data collection period, adequate supervision was undertaken by a supervisor and by a principal investigator. Spot-checking for the collected checklist was done on daily basis. The collected data were checked out for the completeness, accuracy, and clarity by the principal investigator and supervisors.

### 2.2. Study Variables

#### 2.2.1. Dependent Variables

The outcomes of burn injury are the dependent variables.

#### 2.2.2. Independent Variables

Sociodemographic variables include age, sex, and residence. Clinical conditions include surface area of the burn, length of stay, anatomical site of burn, severity (degree) of burn, concomitant medical condition, causes, consequence, prehospital intervention, duration of presentation, complication, nutritional status, and intentional/surgical intervention. Antibiotics, wound care, and fluid resuscitation were also included.

### 2.3. Operational Definitions

The following are the operational definitions of the terms used in the study:

*Outcome of burn injury*: when the children after Hospital management was discharged with complication or without complication as reported on the chart

*Discharge with complication*: when the children were discharged with contracture, disfigurement, amputated, scar of skin graft and death as reported on the chart

*Duration of presentation*: early if the patient comes within or less than 24 hrs and late if the patient comes later than 24 hrs [14]

*Malnutrition*: if there were written same, moderate, and severe malnutrition in the diagnosis part of the children chart at admission time

*Well nutrition*: if there was no written malnutrition in the diagnosis part of children chart at admission time

*Complete card*: if the children's guardian fulfills all questions in the checklist

*Prehospital intervention*: when the children intervened being at a nearby health center or at home

*Discharge without complication*: when the patient was discharged with improvement

### 2.4. Ethical Considerations

Ethical clearance was obtained from Mekelle University College of Health Science Institutional Review Board (IRB). An official letter of permission was written for the administration of Ayder Referral Hospital before the study started, and written consent was obtained from hospital administration to the medical record office. Confidentiality of the medical record was maintained at the time of data collection.

## 3. Result

### 3.1. Sociodemographic Data

A total of 382 hospitalized burn patients were studied from 2011 to 2015, of which 235 (61.5%) patients were male. The mean age of the children was 5.564 years (SD ± 5.423). Two hundred thirty-six (61.8%) burnt children were from an urban area while 146 (38%) were from a rural area. Thirty-five (30.8%) of the burnt children who were discharged with complication were from rural areas.

### 3.2. Clinical Data

#### 3.2.1. Cause of Burn Injury

The most frequent cause of burn was scald; it accounts for 265 (69.4%) of all burn injuries followed by 66 of flame burn (17.3%), 47 of electrical burn (12.3%), 2 of chemical burn (0.5%), and 2 of contact burn (0.5%). Two hundred eight (85%) cases of scald burn occurred within the age group of 0-4 yrs (Table 1).

### 3.3. Extent and Degree of Burn Injuries

**Figure 1:**
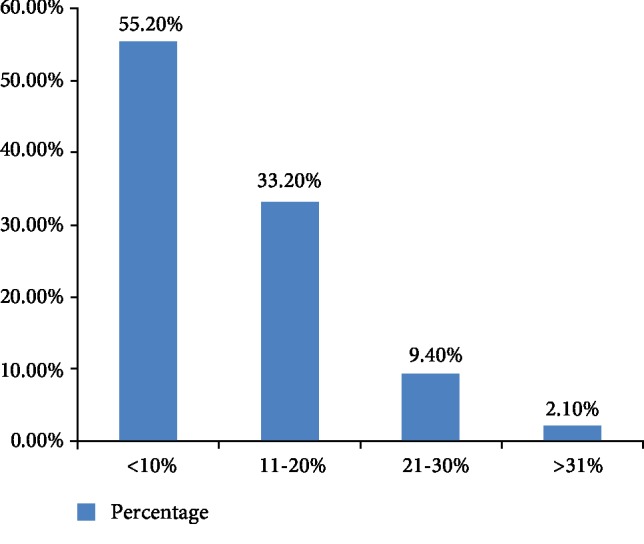
Total body surface area of burned pediatric patients in Ayder Referral Hospital (20112015).

The median total body surface area (TBSA) burned at the time of presentation, determined based on the medical records, was 9% with a range of 1-68% (mean 11.37; SD 8.24). Burn extent in 211 (55.2%) of patients was less than 10% (Figure 1). About six (75%) of burn patients who had a TBSA of >31% were discharged with complication.

Of 382 burn patients, 331 (86.6%) had partial thickness or second-degree burn, 41 (10.7%) had full thickness or third degree, and 10 (2.6%) sustained superficial or first-degree burn. About 28 patients (68.3%) with full thickness were discharged with complication.

### 3.4. Anatomic Locations of Burn Injuries

**Figure 2:**
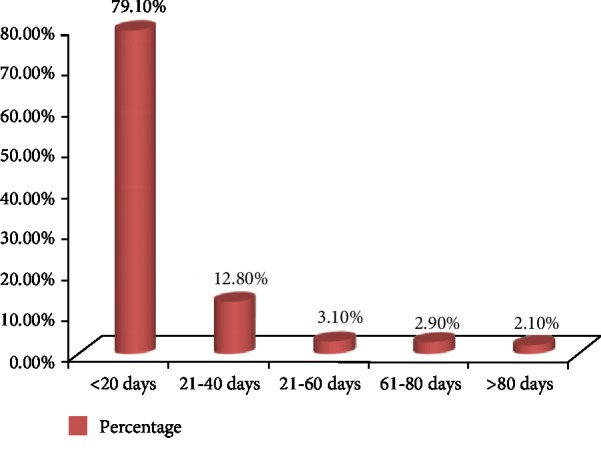
Length of stay of burn pediatric patients in Ayder Referral Hospital (2011-2015).

**Figure 3:**
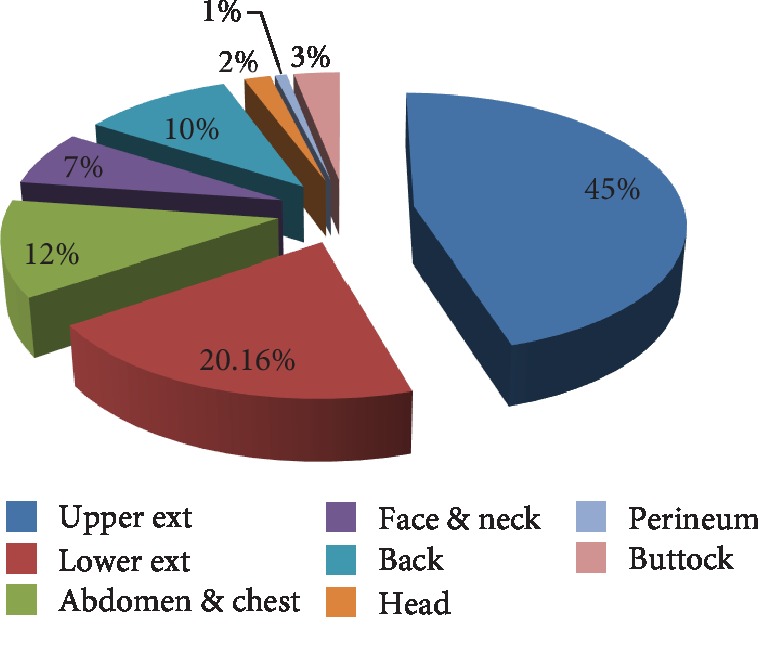
Anatomical location of pediatric burn patients in Ayder Referral Hospital (2011-2015).

Anatomically, the majority of the burns were confined to the upper extremities (173, 45.3%) followed by burns on the lower extremities (77, 20.2%) as shown in Figure 2; patients who sustained burn on their head, face, and neck and upper extremities (42.9%, 34.6%, and 20.8%) were discharged with complication than other parts of the body (Figure 3).

### 3.5. Prehospital Interventions

Among 382 studied burnt patients, prehospital intervention was provided for 202 (52.9%) of patients. Of those patients who had received prehospital interventions at a nearby health institution, there were only 157 (77.7%); the rest of the percentage of preintervention was applied at home; these include cooking oil (4, 2%), coffee powder (14, 7%), call gate (2, 1%), urine and ash (1, 0.5%), and herbs (24, 11.9%). Patients who took prehospital intervention at home 55 (27.2%) were discharged with complication than those who took preintervention (11, 6.1%).

### 3.6. Duration of Hospital Presentation and Length of Stay

Two hundred twenty-seven (59.4%) burn patients were present early (before 24 hours), and 155 (40.6%) burn patients were present lately. The mean length of hospital stay was 17.34 days (SD: 25.334; range: 1-290 days) (Figure 2). Eight (66.7%) patients who stayed from 41 to 60 days were discharged with complication.

### 3.7. Antibiotic, Wound Care, and Fluid Resuscitation

Antibiotic treatment was given to 250 (65.4%) patients; wound care was done in all patients: daily wound care for 328 (85.9%), BID for 51 (13.4%), TID for two (0.5%), and every other day for one (0.3%) patient. Fluid resuscitation was given for only 201 patients (52.6%); lack of fluid resuscitation was given for 44 patients (24.3%) who were discharged with complication than adequate fluid resuscitation within 24 hrs (22, 10.9%).

### 3.8. Early Complication

From the total of pediatric burn patients, 53 (14%) developed early complications like sepsis (3, 0.8%), infection (43, 11.3%), shock (6, 1.6%), and acute pyelonephritis (1, 0.3%), and 329 (86.1%) did not develop early complication. About five (83.3%) patients with shock were discharged with complication.

### 3.9. Management Outcome of Burn Injury

Of the total 382 burn patients, 316 (82.7%) patients were discharged without complication while 66 (17.3%) patients were discharged with complication. Thirty-nine (10.2%) of them had significant morbidity in the form of contractures, 1 (0.3%) developed disfigurement, 6 (1.6%) have amputation of the extremities, 15 (3.9%) constituted scar of skin graft, and five (1.3%) were dead (Table 2).

### 3.10. Factors Associated with Outcome of Burn Injury

In the bivariate analysis, residence, prehospital intervention, duration of presentation, extent of injury, length of stay, age, fluid resuscitation in 24 hours, and nutritional status were found to be statistically significant with burn outcome. After adjusting for the possible confounders in the multivariate analysis, only fluid resuscitation and nutritional status of the burn patient were found to show statistically significant association with outcome of burn injury. Patients with lack of fluid resuscitation within 24 hours had the chance of 2.8 times to be discharged with complication than adequately resuscitated with fluid within 24 hours (AOR = 2.767; 95% CI (1.276-5.999)) and those who were malnourished at admission were 75% protected from being discharged with complication at discharge time (AOR = 0.252; 95% CI (0.069-0.923)) (Table 3).

## 4. Discussion

Burn injury is one of the most devastating and disabling trauma to human being; it remains a serious threat to the well-being of the pediatric population and still has major cosmetic and functional consequences [18], so the purpose of this study was to assess the outcome of burn injury and its associated factors in order to prevent farther morbidity and mortality.

Burns were frequently seen in preschool period and children aged between 0 and 4 yrs [12]. In this study, the highest proportion of burn was seen within the age group of 0-4 yrs (64.1%) which was similar to a study done in Israel (78.0%) [18]. Another two studies conducted in Addis Ababa and Turkey revealed that burns were mostly seen in 0-3 years which were 53.3% (14) and 72.4% [19], respectively. This similarity may be due to the fact that children in this age group have problems with stability and they want to discover their surroundings (environment) as well, and more supervision is essential as the child gains locomotors and manipulative skills that are coupled with in satiable playing around the kitchen and lack of parental supervision which were leading predisposing factors for burns in children of this age group [12].

In this study, 82.7% discharged without complication while 17.3% were discharged with complication. A similar study conducted in Iran showed that about 83% of the patients were discharged with partial recovery, 10% were discharged with complete recovery, and 0.1% referred to other hospitals and 1.36% died [20]. Other observations from Turkey showed that most of the burn patients (93.7%) were discharged from the hospital after receiving treatment. However, 6.3% of the patients died due to burn injury [19]. Another study from South India indicated that 74% of children with burn were discharged with improvement, 6% of the patients LAMA, and 20% died [21]. Another observation conducted in Nigeria showed that 70.1% cases were successfully managed and discharged while 29.9% died [22]. A study from Addis Ababa showed that most of the burns healed with no complication and 33 (7.85%) of the patients died. Among those who died, most of them had flame burn injuries (84.8%) [14]. Most studies used the outcome of burn as discharge with improvement and death, but in this study, discharge with complication and discharge without complication were the outcomes of burn injury; so the researcher compared death from another study and discharge with complication from this study and discharges with improvement the same with other studies. Therefore, the majority of the patients in this study were discharged without complication as the other studies showed above [9, 20–22].

Various observations had reported that scald was the most common cause of burn injuries; a study from Israel showed that scald was the most common type of burn in children (67.4%) [18]. Similar studies done in Morocco reported that scald was the main cause of burn injury (83.5%) [23], a study from Ghana showed 73% of burn was caused by scald [24], another study from Tanzania showed 67.4% of burn was caused by scald burn [25]. Similarly, in this study, the most frequent cause of burn injuries was scald (69.4%). This can be explained by the fact that children especially toddlers and preschool children stay with their mothers or caregivers at home and would probably be left playing in the kitchen environment; as a result, they can get scalded by liquid foods being cooked by their parents or caregivers.

The total body surface area (TBSA (%)) affected due to burn injury is an important factor in the determination of the burn injury. In this study, majority (55.2%) of the patients had burn extent of less than 10% of TBSA. The mean TBSA was 11.37%. This finding is a bit smaller than a study conducted in Turkey and Addis Ababa in which the overall mean total burn surface area (TBSA) was 14.1 with the range of 1-88 days and 11 with a range of 1-95% (mean 14.7; SD 12.3), respectively. The reason that this study reported smaller mean or median of TBSA from the findings of Turkey and Addis Ababa may be due to the difference in the admission criteria and the performance of the health care providers.

In this study, the upper extremities (45.3%) were the most affected area followed by lower extremities (20.2%); this finding was similar with a study conducted in Turkey in which 71.8% of the burns occur in the upper extremities [19]. The upper limbs were the most involved in 90.42% of the cases followed by the lower limbs (80.85%), as the study from Nigeria showed [22]. Studies may be attributable to the similarity of the causative factors. Scalding was the most frequently occurred when children reached for a container of hot liquid while playing, by pulling a hot substance from either a cooking stove or a countertop. This could result in immersion or spilling of the hot substance on the children's extremities.

In this study, lack of fluid resuscitation within 24 hours was 2.8 times discharged with complication than adequate fluid resuscitated within 24 hours (AOR = 2.767; 95% CI (1.276-5.999)). Similarly, a study conducted in Iran showed that lack of fluid resuscitation before hospital has positive relationship with mortality of their patients (AOR = 40.5, 95% CI (10.2-51.55)). Unlike this study, a study from the United States showed that 98% of children were resuscitated successfully, with 24 hours. This difference may be because 40.6% of burn patients in this study were admitted late (after 24 hrs). A study conducted in Brazil showed that 61% of patients were diagnosed with malnutrition. These patients had a significantly longer delay to transfer for definitive care (26–166 days) than well-nourished patients who transferred at 21–138 days (*P* < 0.05). In contrast, this study showed that those who were malnourished patients at admission were 75% protected from being discharged with complication at discharge time (AOR = 0.252, 95% CI (0.069-0.923)). This may be because there is a dietitian from a board in the hospital and she offered them a special feeding according to their needed calorie in addition to hospital feeding and therapeutic feeding; this may make them have a good outcome at discharge time.

## 5. Conclusion

In conclusion, majority of the pediatric burn patients were discharged without complication. The most causative agent of these accidents was scald; upper extremities also were the most affected area. The factors associated with outcome of burn injury according to this study were lack of fluid resuscitation and malnourishment of burn patients.

Based on the finding of the result in this study, the following recommendations are forwarded: for the hospital, since majority of the malnourished burn patients had good outcome, the hospital management should maintain offering them special feeding forever without interruption.

For the health professionals, lack of fluid resuscitation within 24 hrs makes the patients being discharged with complication; therefore, health education should be given by health professionals especially by health extension workers to the parents or caregivers (community) to come as early as possible as soon as the accident or the injury occurs.

For researchers, it will be valuable if further studies will be conducted to assess the standard management trends of pediatric burns since the same patients who were discharged with complication could possibly undergo inadequate resuscitation.

It will be better if further studies will be conducted prospectively to assess burn patients, how they were burned, who was with them, what was their parents economic status, how was their housing condition, and the like or to get comprehensive information.

## Figures and Tables

**Table 1:** Cause of burn trauma in different age groups of pediatric patients in Ayder Referral Hospital (2011-2015).

Variable	Category	Cause of burn injury					Total
		Scald	Flame	Electrical	Chemical	Contact	
Age	<4 yrs	208 (84.9%)	31 (12.7%)	4 (1.6%)	0 (0%)	2 (0.8%)	245 (100%)
	5-9 yrs	32 (62.7%)	11 (21.6%)	8 (15.7%)	0 (0%)	0 (0%)	51 (100%)
	10-14 yrs	15 (37.5%)	9 (22.5%)	15 (37.5%)	1 (2.5%)	0 (0%)	40 (100%)
	>15	10 (21.7%)	15 (32.6%)	20 (43.5%)	1 (2.2%)	0 (0%)	46 (100%)
Total		265 (69.4%)	66 (17.3%)	47 (12.3%)	2 (0.5%)	2 (0.5%)	382 (100%)

**Table 2:** Outcome of burn injury by age of pediatric burn patients in Ayder Referral Hospital (20112015).

Variables	Category	Consequence						Total
		Developed contracture	Developed disfigurement	Amputated	Scar of skin graft	Death	None	
Age group	<4 yrs	22	1	2	3	2	215	245
	5-9.5 yrs	7	0	0	3	1	40	51
	10-14 yrs	5	0	1	6	0	28	40
	>15 yrs	5	0	3	3	2	33	46
Total		39	1	6	15	5	316	382

**Table 3:** Binary logistic regression analysis of outcome of burn with sociodemographic and clinical data in Ayder Referral Hospital burn unit, Mekelle city, Tigray region, Ethiopia, 2015/16.

Factor	Outcome of burn injury			COR	95% CI	AOR	95% CI
	Dis. with complication		Dis. without complication				
	No. (%)		No. (%)				
Residence							
Urban	21 (8.9)		215 (91.1)	**1.00**			
Rural	45 (30.8)		101 (69.2)	0.219	**(0.124-0.387)** ^∗^	1.463	(0.655-3.266)
Total	66 (17.3)		316 (82.7)				
Prehospital intervention							
Yes	55 (27.2)		147 (72.8)	**1.00**			
No	11 (6.1)		169 (93.9)	0.174	**(0.088-0.345)** ^∗^	0.626	(0.234-1.673)
Total	66 (17.3)		316 (82.7)				
Duration of presentation							
Early	13 (5.7)		214 (94.3)	**1.00**			
Late	53 (34.2)		102 (65.8)	**8.554**	(**4.462-16.398**)^∗^	1.973	(0.710-5.484)
Total	66 (17.3)		316 (82.7)				
Extent	25 (11.8)		186 (88.2)	1.00			
<10%							
11-20%	20 (15.7)		107 (84.3)	0.719	(0.831-1.356)	2.080	(0.289-14.995)
21-30%	15 (41.7)		21 (58.3)	0.188	**(0.086-0.412)** ^∗^	**1.850**	**(0.257-13.325)**
>31%	6 (75)		2 (25)	**0.045**	**(0.009-0.234)** ^∗^	**1.492**	**(0.204-10.888)**
Total	66 (17.3)		316 (82.7)				
Length of stay							
<20 days		32 (10.6)	270 (89.4)	**1.00**			
21-40 days		16 (32.7)	33 (67.3)	**0.244**	**(0.121-0.493)** ^∗^	3.454	(0.638-18.699)
41-60 days		8 (66.7)	4 (33.3)	**0.059**	**(0.017-0.208)** ^∗^	1.027	(0.181-5.817)
61-80 days		6 (54.5)	5 (45.5)	**0.099**	**(0.029-0.342)** ^∗^	0.277	(0.035-2.207)
>81 days		4 (50)	4 (50)	**0.119**	**(0.028-0.497)** ^∗^	0.767	(0.093-6.294)
Total	66 (17.3) **fluid**		316 (82.7)				
Resuscitation in 24 hrs							
Yes	22 (10.9)		179 (89.1)	**1.00**			
No	44 (24.3)		137 (75.7)	2.6132	**(1.496-4.566)** ^∗^	2.767	**(1.276-5.999)** ^∗∗^
Total	66 (17.3)		316 (82.7)				
Nutritional status at admission							
Malnutrition	7 (50)		7 (50)	0.191	**(0.65-0.565)** ^∗^	0.252	**(0.069-0.923)** ^∗∗^
Well nutrition	59 (16)		309 (84)	**1.00**			
Total	66 (17.3)		316 (82.7)				
Age							
<4.5years		32 (12.8)	218 (87.2)	0.603	(0.266-1.376)	0.599	(0.178-2.019)
5-9.5 years		9 (19.6)	37 (80.4)	0.343	**(0.158-0.741)** ^∗^	1.125	(0.295-4.295)
10-14.5years		12 (30)	28 (70)	0.73	**(0.178-0.782)** ^∗^	0.402	(0.116-1.389)
>15 years		13 (28.3)	33 (71.7)	1.00			
Total		66 (17.3)	316 (82.7)				

^∗^Dis: discharge. ^∗^Significant association in bivariate analysis. ^∗∗^Significant association in multivariable analysis.

## Data Availability

The datasets used and/or analyzed during the current study are available from the corresponding author on reasonable request.
